# Effects of novel flame retardants tris(1,3-dichloro-2-propyl) phosphate (TDCIPP) and triphenyl phosphate (TPhP) on function and homeostasis in human and rat pancreatic beta-cell lines

**DOI:** 10.1007/s00204-024-03841-z

**Published:** 2024-08-27

**Authors:** Nela Pavlíková, Jan Šrámek, Vlasta Němcová, Lola Bajard

**Affiliations:** 1 3LF UK, Departement of Biochemistry, Cell and Molecular Biology & Center for Research On Nutrition, Metabolism, and Diabetes, Third Faculty of Medicine, Charles University, Ruska 87, 100 00, Prague, Czech Republic; 2 Faculty of Science, RECETOX, Masaryk University, Kotlarska 2, 611 37 Brno, Czech Republic

**Keywords:** TDCIPP, TPhP, Diabetes, Beta-cells, Insulin, Metabolic disease

## Abstract

Despite the fact that environmental pollution has been implicated in the global rise of diabetes, the research on the impact of emerging pollutants such as novel flame retardants remains limited. In line with the shift towards the use of non-animal approaches in toxicological testing, this study aimed to investigate the effects of two novel flame retardants tris(1,3-dichloro-2-propyl) phosphate (TDCIPP) and triphenyl phosphate (TPhP) in rat (INS1E) and human (NES2Y) pancreatic beta-cell lines. One-week exposure to 1 μM and 10 μM TDCIPP and TPhP altered intracellular insulin and proinsulin levels, but not the levels of secreted insulin (despite the presence of a statistically insignificant trend). The exposures also altered the protein expression of several factors involved in beta-cell metabolic pathways and signaling, including ATP citrate lyase, isocitrate dehydrogenase 1, perilipins, glucose transporters, ER stress-related factors, and antioxidant enzymes. This study has brought new and valuable insights into the toxicity of TDCIPP and TPhP on beta-cell function and revealed alterations that might impact insulin secretion after more extended exposure. It also adds to the scarce studies using in vitro pancreatic beta-cells models in toxicological testing, thereby promoting the development of non-animal testing strategy for identifying pro-diabetic effects of chemical pollutants.

## Introduction

Environmental pollution has become an admitted factor in the development of worldwide diabetes epidemics (Hoyeck et al. 2022). For instance, multiple reviews mostly based on epidemiological or in vivo animal studies describe persistent organic pollutants (Kim et al. 2019; Lee et al. 2018), air pollution (Yang et al. 2020), or pollutant-affected gut microbiota (Bailey et al. 2020) to play a role in the development of type-2 diabetes mellitus. Data also exist connecting persistent organic pollutants and type-1 diabetes (Bresson et al. 2022). A recent review highlights the associations between exposure to several environmental pollutants, such as phthalates or polybrominated diphenyl ethers (PBDEs), and the development of gestational diabetes mellitus (Yao et al. 2023). Among the possible mechanisms involved, increased oxidative stress in pancreatic beta-cells (Hoyeck et al. 2022; Jiang et al. 2021; Loiola et al. 2016; Marroqui et al. 2018; Park et al. 2020) or decreased insulin production (Hoyeck et al. 2022; Pavlikova et al. 2019, 2023) emerge from the literature in connection with type 2 diabetes development. However, studies remain scarce for many emerging pollutants, such as novel flame retardants (FRs).

FR chemicals are added to consumer products to delay flammability but may pose public health concerns. For instance, the long-used brominated FRs, such as PBDEs, have been added to Annex A of the Stockholm Convention on persistent organic pollutants, meaning they should be eliminated from the market. To meet the flammability standards, novel FRs, such as organophosphorus FRs (OPFRs), are being used as replacements. However, an increasing number of studies point to possible health effects (such as endocrine disruption, male reproductive disorders, hepatotoxicity, or neurotoxicity) associated with exposure to several OPFRs (Bajard et al. 2019, 2021; Blum et al. 2019b; Dishaw et al. 2014; Zhao et al. 2022). In particular, a few reports indicate the possible effects of novel FRs with type 2 diabetes mellitus. For instance, exposure to triphenyl phosphate (TPhP) reduces insulin-induced glucose uptake by liver cell lines and induces insulin resistance in mice (Yue et al. 2023). TPhP exposure in mice fetus also impacts glucose clearance (Wang et al. 2019). Another two studies in mice show that exposure to a mixture of tris(1,3-dichloro-2-propyl) phosphate (TDCIPP), TPhP, and tricresyl phosphate may alter plasma insulin levels and glucose clearance in a sex- and diet-dependent way (Krumm et al. 2018; Walley et al. 2021). These limited studies alert on the possible impact of OPFRs on glucose homeostasis and the development of type 2 diabetes mellitus. However, more studies are required to confirm these effects and elucidate the underlying toxicological mechanisms.

For more than a decade, alternative approaches to animal testing, such as in vitro cell models, have been strongly encouraged and increasingly employed in toxicological studies due to ethical, efficacy, and financial issues (Krewski et al. 2010; Langley et al. 2007). In particular, in vitro cultures are largely used to study the ability of chemicals to induce hepatotoxicity (Gómez-Lechón MJ et al. 2014; Negi et al. 2021) or skin toxicity (Hoffmann et al. 2018; Kleinstreuer et al. 2018). Utilizing beta-cell lines is crucial to address sustainability and ethical concerns associated with using primary human islets from donors. However, in vitro systems for the pancreas remain remarkably limited. Currently, no in vitro model for Langerhans islets exists, and the islets used in ex vivo experiments are directly isolated from rodents or humans. In vitro systems used for diabetes research consist of a few pancreatic beta-cell lines available.

This study aimed to test the effects of TDCIPP and TPhP on a rat beta-cell line (INS1E) and a human pancreatic beta-cell line (NES2Y). After a one-week exposure, we examined the expression of proteins involved in insulin production, glucose transport, metabolism, and oxidative stress defense.

## Materials and methods

### Materials

TDCIPP (CAS# 13,674-87-8) was purchased from Tokyo Chemical Industry (TCI) (> 95% purity, Cat# P0269, Lot# EFBGE-LQ), and TPhP (CAS# 115-86-6) from Sigma-Aldrich (> 99% purity, Cat# 24–12-88, Lot# BCBW9556) and RPMI medium from Sigma-Aldrich (https://www.sigmaaldrich.com). We purchased primary antibodies for aconitase 2 (ab129069), ATP citrate lyase (ab40793), isocitrate dehydrogenase 1 (ab172964), glutathione reductase (ab16801), superoxide dismutase 1 (ab51254), binding immunoglobulin protein (ab21685), phosphorylated iron-responsive element 1 (S724) (ab48187), and hypoxanthine–guanine phosphoribosyltransferase (ab109021) from Abcam (www.abcam.com); primary antibodies for biliverdin reductase B (17,729-1-AP), glucose transporter 1 (121,829-1-AP), glucose transporter 2 (20,436-1-AP), perilipin 2 (15,294-1-AP), and perilipin 5 (26,951-1AP) from Fisher Scientific (https://www.thermofisher.cz); primary antibody for cyclic AMP-dependent transcription factor (HPA001562), and actin (a3853) from Sigma-Aldrich (https://www.sigmaaldrich.com); and primary antibody for phosphorylated protein kinase A (Thr197) from Cell Signaling (https://www.cellsignal.com). We used Pierce^™^ BCA Protein Assay Kit (https://www.thermofisher.cz) to determine protein concentrations.

### Cell culture

Our experiments employed the rat pancreatic beta-cell line INS1E with glucose-stimulated insulin secretion (kindly provided by Dr. Claes B. Wollheim at the Centre Medical Universitaire de Genève, Geneva, Switzerland) and the human pancreatic beta-cell line NES2Y with constitutive insulin secretion (kindly provided by Dr. Roger F. James, Department of Infection, Immunity and Inflammation, University of Leicester). We used a medium based on RPMI 1640, containing phenol red, L-glutamine, sodium pyruvate, HEPES, penicillin, and streptomycin, and supplemented with 10% fetal bovine serum (FBS), as previously described (Pavlikova et al. 2015) for culturing the cells. We maintained cells in a humidified atmosphere of 5% CO_2_ in air at 37 °C.

### Viability assessment (neutral red assay)

We performed a Neutral Red Assay to determine the viability of pancreatic beta-cells exposed for 48 h to the following concentrations of TDCIPP or TPhP: 10 nM, 100 nM, 1 μM, 10 μM, and 100 μM, as described before (Pavlikova et al. 2023).

### One-week exposure to pollutants

We seeded beta-cells into three six-well plates using a density of 50 000 cells/well/3 ml of media for NES2Y cells and 150 000 cells/well/3 ml for INS1E cells. The media already contained the selected concentrations (1 μM or 10 μM) of pollutants (TDCIPP or TPhP) or DMSO as solvent control (the concentration of DMSO in the medium was 0.5%). After 4 days, we replaced the medium with a new one containing pollutants. After three more days (7 days total), we removed the medium, washed cells three times with PBS, added RIPA lysis buffer containing protease and phosphatase inhibitors, and stored the plates at −80 °C. The next day, we thawed the contents of the wells and moved them to Eppendorf tubes. After centrifuging the lysates, we transferred the supernatants into new Eppendorf tubes and stored them at −80 °C. The protein concentrations were quantified employing the BCA commercial kit.

### Western blot

We performed western blots as described before (Pavlikova et al. 2023) with minor modifications. We separated 18 ug of proteins on 10% polyacrylamide gels and then blotted them onto a nitrocellulose membrane. We incubated membranes with the primary antibodies overnight at 4 °C on a shaker. We used primary antibodies at concentrations 1:1000 for all primary antibodies except for HPRT and BiP, where we used concentration 1:10,000. Primary antibodies against phosphorylated forms of proteins were diluted in 5% BSA; the rest of the primary antibodies were diluted in 5% low-fat milk.

### ELISA: intracellular insulin

We employed commercial kits to determine intracellular insulin and proinsulin levels (https://www.mercodia.com/; 10-1232-01, 10-1250-01). We diluted our samples into 1 μg/μl stock solutions. We used those stock solutions for further dilution of samples to find the concentrations fitting into the calibration curve. After performing the ELISA experiment, we determined the protein concentration of the 1 μg/μl sample solutions and normalized the results from ELISA to those values.

### ELISA: insulin secretion

We seeded INS1E cells into a 24-well plate at a density of 50 000 cells/well/750 μL. The media already contained the selected concentrations (1 μM or 10 μM) of pollutants (TDCIPP or TPhP) or DMSO as solvent control. After 7 days of exposure, we determined insulin secretion. We removed the medium, washed the cells with PBS, and added RPMI medium without glucose for 2 h. After 2 h, we removed the RPMI medium and added Krebs buffer containing glucose 2.5 mM, glucose 25 mM, or KCl 30 mM combined with glucose 2.5 mM. After 30 min, we collected the Krebs buffer, centrifuged it (5000 g/5 min), and determined insulin content using a commercial kit (https://www.mercodia.com/; 10-1250-01).

### Use of large language models

During the preparation of this work, the authors used GPT Poe to improve the manuscript’s language. After using this tool, the authors reviewed and edited the content as needed and take full responsibility for the content of the publication.

## Results

### Cell viability

**Fig. 1:**
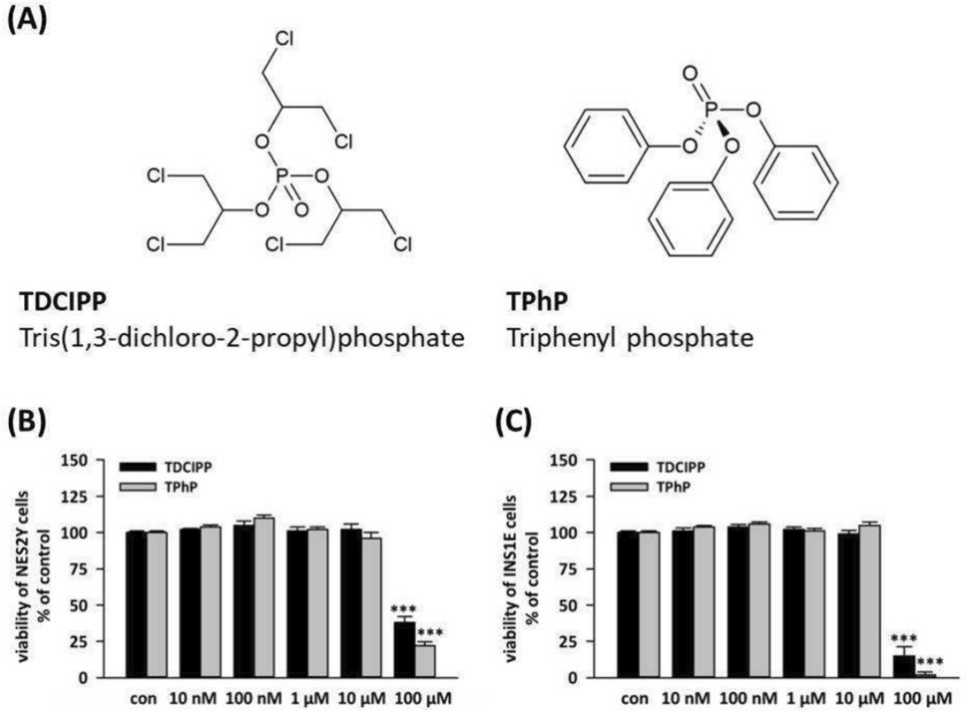
shows the chemical structures of TDCIPP and TPhP (**A**), the viability of (**B**) rat pancreatic beta-cells INS1E and (**C**) human pancreatic beta-cells NES2Y when exposed to DMOSO (con), 10 nM, 100 nM, 1 μM, 10 μM, and 100 μM of TDCIPP and TPhP for 48 h detected by a Neutral Red Assay. The graph shows the average of three independent experiments ± SEM. *** means statistical significance (*p * <0.001) determined by ONE-WAY ANOVA (Dunnet’s test)

From the tested concentrations (10 nM, 100 nM, 1 μM, 10 μM, and 100 μM), only 100 μM of both flame retardants affected beta-cell viability measured using the Neutral Red Assay. After 48-h exposure, 100 μM TDCIPP significantly decreased INS1E cell viability (Fig. 1B) to 15% of control and NES2Y cell viability (Fig. 1C) to 38% of control; 100 μM TPhP significantly decreased INS1E cell viability (Fig. 1B) to 2% of control and NES2Y cell viability (Fig. 1C) to 22% of control. For the follow-up examination of effects on cell functions in one-week exposure, we chose the two highest non-cytotoxic concentrations from our scale: 1 μM and 10 μM.

### Proinsulin and intracellular insulin levels and insulin secretion

**Fig. 2:**
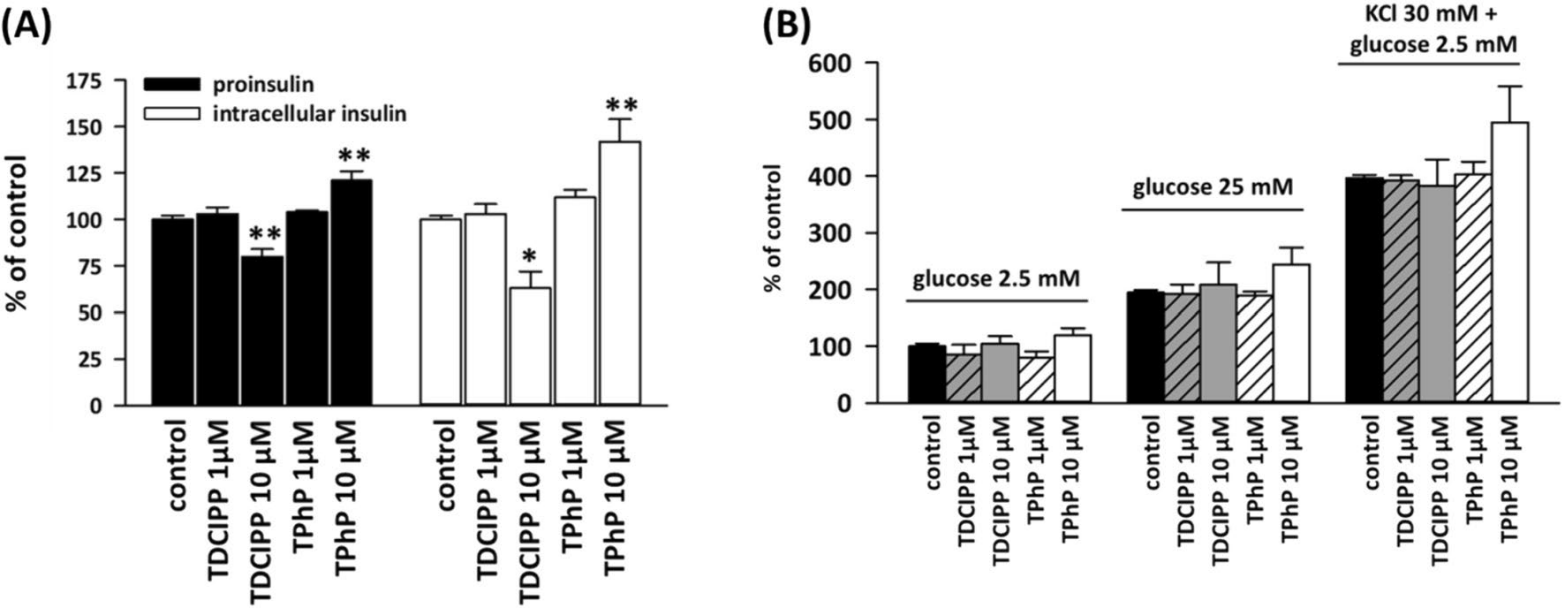
**A** Shows the levels of intracellular insulin and proinsulin in rat pancreatic beta-cells INS1E exposed to 1 μM and 10 μM TDCIPP and TPhP for one week detected by ELISA. **B** Shows insulin secretion by rat pancreatic beta-cells INS1E exposed to 1 μM and 10 μM concentrations of TDCIPP and TPhP for 1 week detected by ELISA. The graph shows the mean of four independent experiments (2**A**) and two independent experiments (2**B**) ± SEM. ** means statistical significance (*p* < 0.01), * means statistical significance (*p* < 0.05) determined by ONE-WAY ANOVA (Dunnet’s test)

In INS1E cells, both TDCIPP and TPhP (Fig. 1A) significantly affected intracellular levels of proinsulin and insulin but had opposing effects (Fig. 2A). One-week exposure to 10 μM TDCIPP decreased proinsulin level to 80% of control and intracellular insulin level to 63% of control. On the other hand, 1-week exposure to 10 μM TPhP increased proinsulin level to 121% of control and intracellular insulin level to 142% of control.

We then measured secreted insulin in INS1E cells. We have tested insulin secretion of the exposed cells stimulated by 2.5 mM glucose (hypoglycemia), 25 mM glucose (hyperglycemia), and 2.5 mM glucose in combination with 30 mM KCl, which stimulates insulin secretion independently on glucose intake. The exposure to pollutants did not significantly affect the levels of secreted insulin (Fig. 2B). Consistent with the effects on intracellular insulin, the exposure to 10 μM TPhP increased secreted insulin level when stimulated by high glucose concentration or KCl, but the changes were insignificant (Fig. 2B).

### Effects on enzymes related to NADPH production

NADPH stimulates insulin secretion independently of the main glucose-stimulated signaling pathway (glucose uptake leads to increased ATP level and potassium channel closure resulting in voltage-gated calcium channel opening) by interaction with the insulin vesicle secretory mechanism, thereby amplifying the secretion process (Campbell and Newgard 2021; Zhang et al. 2021). The precise mechanism behind this phenomenon is unknown, but it was confirmed also in human beta-cells obtained from non-diabetic and type 2 diabetic donors (Ferdaoussi et al. 2015). Isocitrate dehydrogenase 1 (IDH1) generates NADPH from isocitrate in the cytosol, while aconitase-2 acts prior to IDH in the tricarboxylic acid cycle, facilitating the conversion of citrate to isocitrate.

**Fig. 3:**
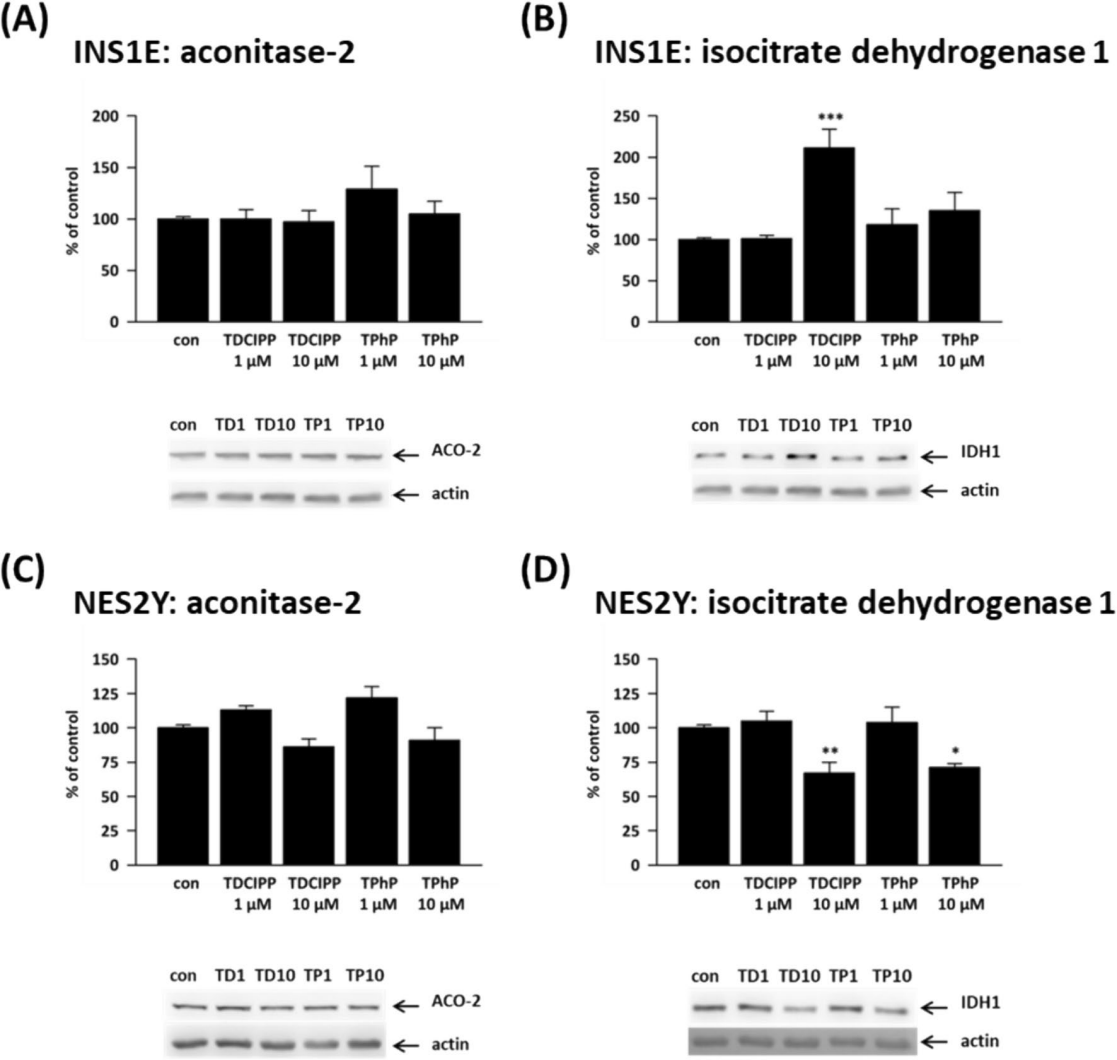
Shows the protein expression of (**A**, **C**) aconitase-2 (ACO-2) and (**B**, **D**) isocitrate dehydrogenase 1 (IDH1) in the rat (INS1E) pancreatic beta-cells and human (NES2Y) pancreatic beta-cells exposed to 1 μM and 10 μM TDCIPP and TPhP for 1 week. Actin was used as a loading control. The graphs represent an average of densitometric analyses of at least four western blots made of four independent sets of samples ± SEM. Below each graph, a representative western blot is shown. * means statistical significance (*p* < 0.05), ** means statistical significance (*p<* 0.01), *** means statistical significance (*p* < 0.001) determined by ONE-WAY ANOVA (Dunnet’s test)

The effects of flame retardants on IDH1 expression differed between rat and human beta-cells. In rat beta-cells (Fig. 3B), 10 μM TDCIPP significantly increased IDH1 protein expression to 211% of the control; 10 μM TPhP exposure showed no effect. In human beta-cells (Fig. 3D), both 10 μM TDCIPP and 10 μM TPhP significantly decreased IDH-1 protein expression to 67% and 71% of the control, respectively.

### .

Neither TPhP nor TDCIPP significantly altered the protein expression of aconitase-2 (ACO-2) in beta-cells (Fig. 3A, C), but there was a decreasing trend at 10 μM concentrations in human beta-cells (Fig. 3C).

### Proteins involved in ER stress and translation

ER stress in pancreatic beta-cells leads to decreased insulin production (Fonseca et al. 2010; Wang et al. 2016). In response to ER stress, the cells initiate unfolded protein response (UPR), the chain of reactions that helps ER manage the accumulated unfolded proteins (Gong et al. 2017). The UPR comprises several branches; we tested the BiP–PERK–p-eIF2α branch and p-IRE1α branch (Cnop et al. 2017).

**Fig. 4:**
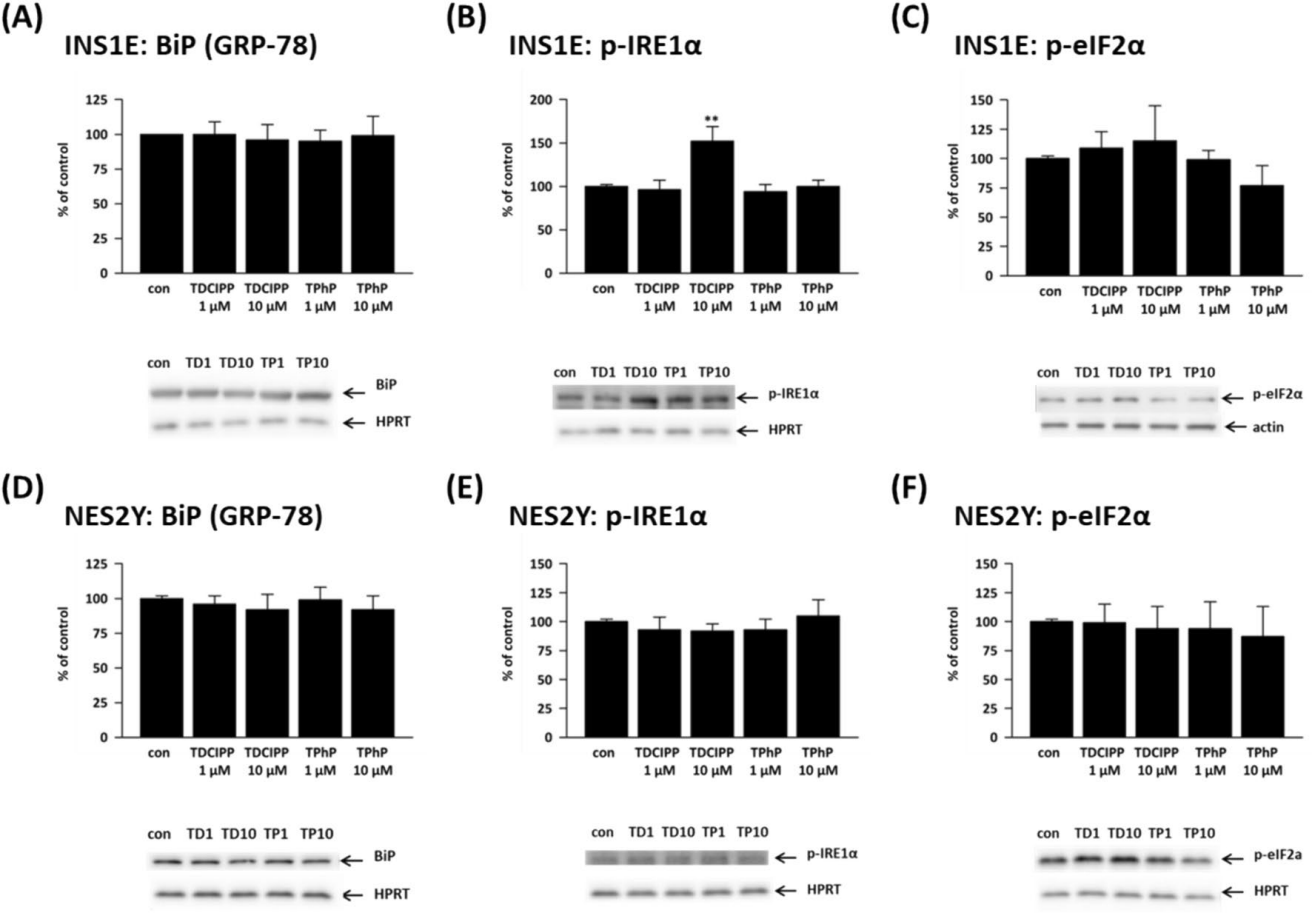
Shows the protein expression of (**A**, **D**) binding immunoglobulin protein (BiP/GRP-78), (**B**, **E**) inositol-requiring enzyme 1α (p-IRE1α), and (**C**, **F**) phosphorylated eukaryotic initiation factor 2 (p-eIF2α) in the rat (INS1E) pancreatic beta-cells and human (NES2Y) pancreatic beta-cells exposed to 1 μM and 10 μM TDCIPP and TPhP for 1 week. Actin or HPRT was used as a loading control. The graphs represent an average of densitometric analyses of at least four western blots made of three independent sets of samples ± SEM. Below each graph, a representative western blot is shown. ** means statistical significance (*p* < 0.01) determined by ONE-WAY ANOVA (Dunnet’s test)

One-week exposure to flame retardants failed to alter the protein expression of BiP in both rat (Fig. 4A) and human beta-cells (Fig. 4D). The exposure to both chemicals (their 10 μM concentrations) altered the levels of the phosphorylated form of eIF2α (p-eIF2α), but the changes were inconsistent and not statistically significant in any cell line (Fig. 4C and 4F). In rat beta-cells, 10 μM TDCIPP significantly increased the phosphorylated form of IRE1α to 152% of control (Fig. 4B); in human beta-cells, p-IRE1α remained unchanged (Fig. 4E).

### Glucose transporters

Glucose transporters (GLUT) facilitate the transport of extracellular glucose into the cells. They represent the first step of glucose metabolism, which eventually leads to insulin secretion.

**Fig. 5:**
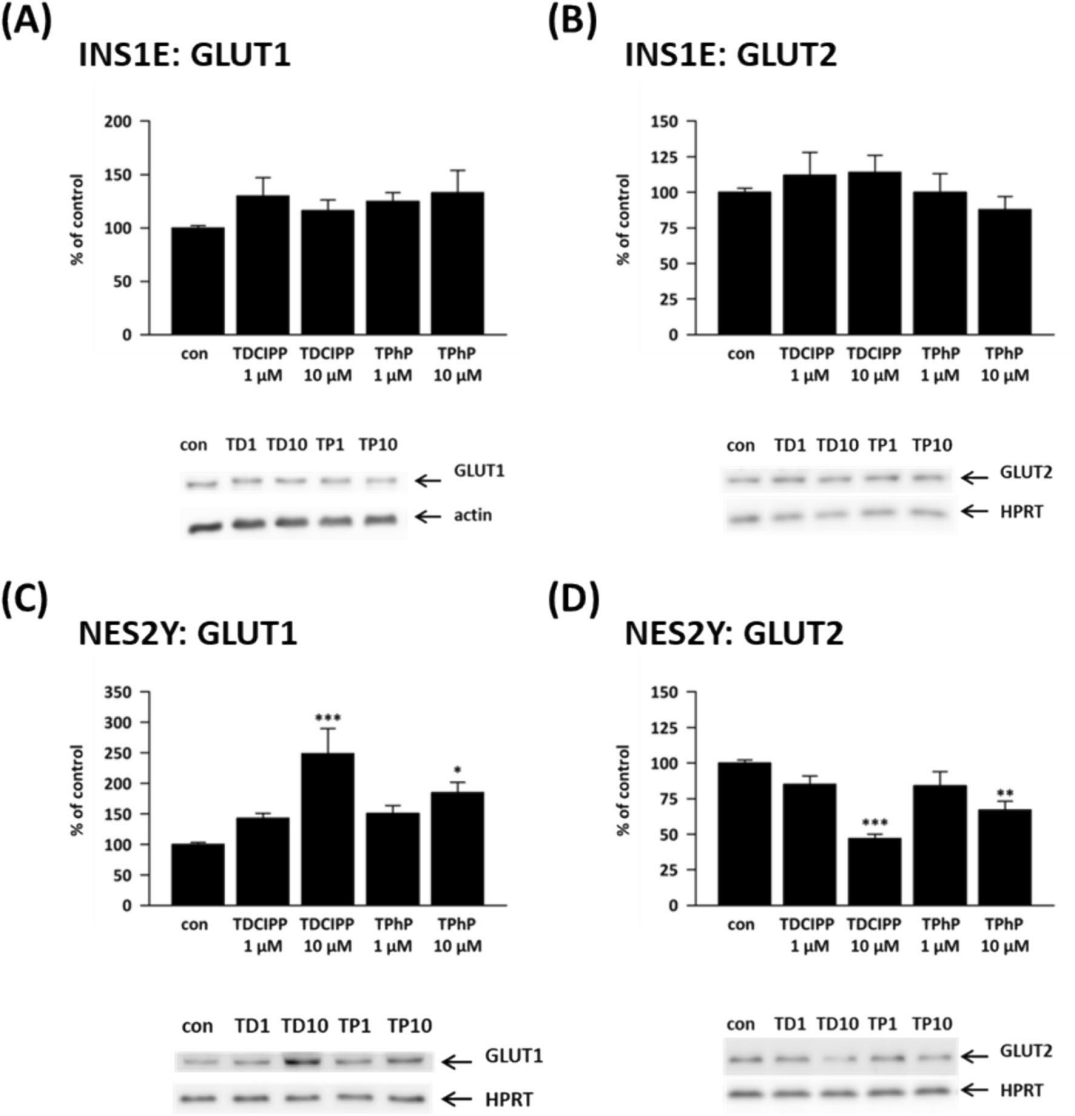
The protein expression of (**A**, **C**) glucose transporter 1 (GLUT1) and (**B**, **D**) glucose transporter 2 (GLUT2) in the rat (INS1E) and human (NES2Y) pancreatic beta-cells exposed to 1 μM and 10 μM TDCIPP and TPhP for 1 week. Actin or HPRT was used as a loading control. The graphs represent the mean ± SEM of densitometric analyses of at least four western blots from four independent sets of samples. Below each graph, a representative western blot is shown ± SEM. *means statistical significance (*p* < 0.05), **means statistical significance (*p* < 0.01), *** means statistical significance (*p* < 0.001) determined by ONE-WAY ANOVA (Dunnet's test)

In rat beta-cells, exposure to pollutants failed to alter the protein expression of GLUT1 or GLUT2 (Fig. 5A and 5B). In human beta-cells, 10 μM TDCIPP increased GLUT1 protein expression to 249% of control (Fig. 5C) and decreased GLUT2 protein expression to 47% of control (Fig. 5D); both changes were statistically significant. In human beta-cells, 10 μM TPhP increased GLUT1 protein expression to 185% of control (Fig. 5C) and decreased GLUT2 protein expression to 67% of control; both changes were statistically significant. A consistent but insignificant trend was observed for both chemicals at 1 µM.

### Proteins involved in lipid synthesis and lipid droplet formation

ATP citrate lyase (ATP-CL) plays a critical role in cellular lipid production. It acts as a bridge between glucose and fatty acid metabolism by converting citrate into acetyl-CoA in the cytosol. Acetyl-CoA is a substrate for forming malonyl-CoA, which can stimulate insulin secretion (Aghelan et al. 2020).

**Fig. 6:**
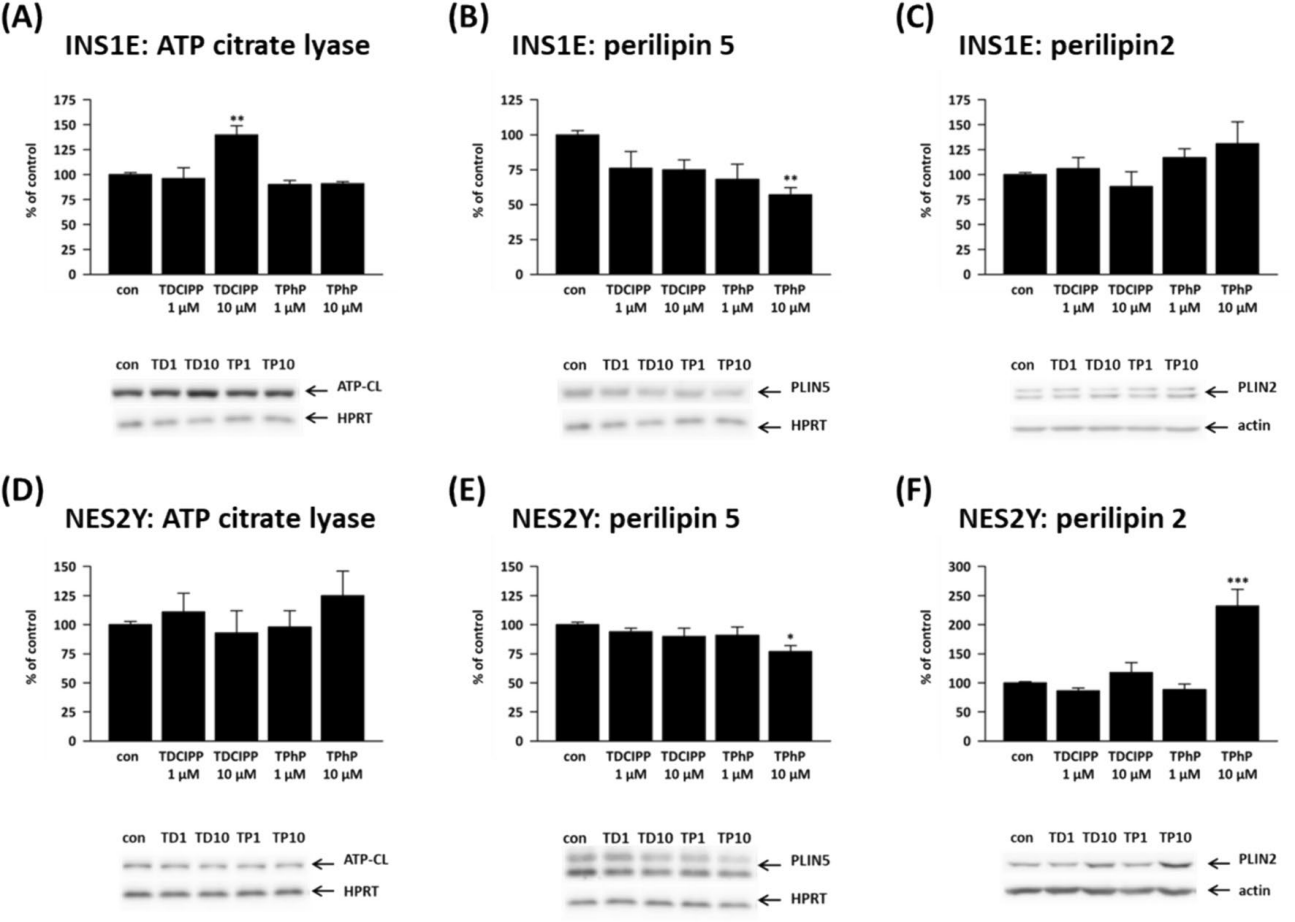
The protein expression of (**A**, **D**) ATP citrate lyase (ATP-CL), (**B**, **E**) perilipin 5 (PLIN5) and (**C**, **F**) perilipin 2 (PLIN2) in the rat (INS1E) pancreatic beta-cells and human (NES2Y) pancreatic beta-cells exposed to 1 μM and 10 μM concentrations of TDCIPP and TPhP for 1 week. Actin or HPRT was used as a loading control. The graphs represent an average of densitometric analyses of at least four western blots made of four independent sets of samples ± SEM. Below each graph, a representative western blot is shown. *means statistical significance (*p* < 0.05), ** means statistical significance (*p* < 0.01), ***means statistical significance (*p* < 0.001) determined by ONE-WAYANOVA (Dunnet's test)

One-week exposure to 10 μM TDCIPP significantly increased protein expression of ATP citrate lyase (ATP-CL) to 140% of control in rat (Fig. 6A) but not human beta-cells (Fig. 6D).

Perilipins play an essential role in lipid droplet formation in a reaction to nutrition overload. In adipocytes, pollutants like DDE (Mullerova et al. 2016), methyl-Hg (Tinant et al. 2021), or PCB-138 (Kim et al. 2018) altered the expression of perilipins and acted as obesogens. Our study focused on two perilipins present in pancreatic beta-cells: perilipin 2 and perilipin 5.

10 μM TPhP decreased protein expression of perilipin 5 (PLIN5) in both beta-cell lines to 57% of control in rat beta-cells (Fig. 6B) and 77% of control in human beta-cells (Fig. 6E). Interestingly, in human (but not rat, Fig. 6C) beta-cells, the TPhP-induced PLIN5 downregulation is accompanied by a significant perilipin 2 (PLIN2) upregulation to 232% of control (Fig. 6F).

### Proteins related to cAMP signaling

**Fig. 7:**
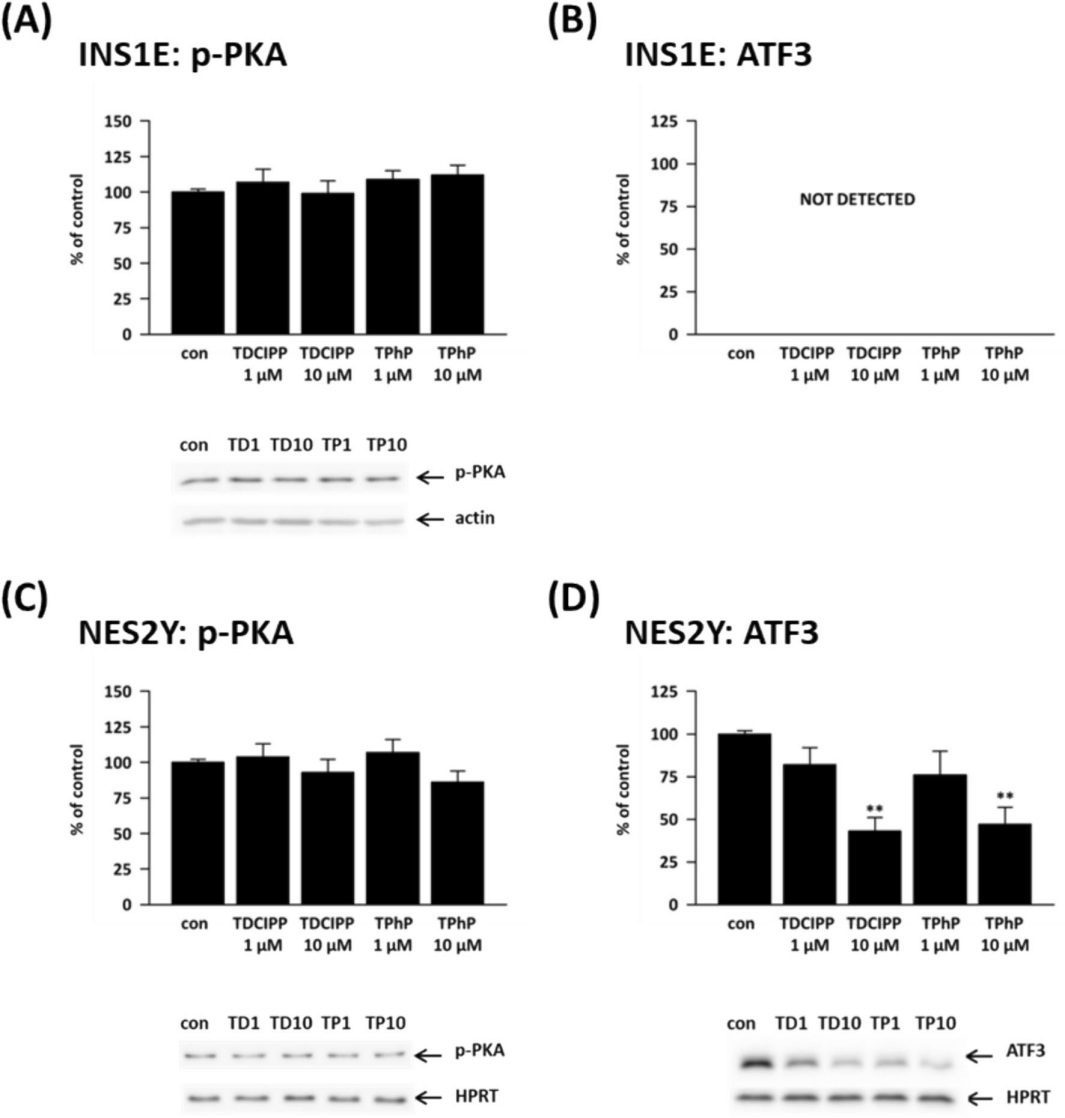
Shows the protein expression of (**A**, **C**) phosphorylated form of protein kinase A (p-PKA) and (**B**, **D**) activating transcription factor 3 (ATF3) in the rat (INS1E) pancreatic beta-cells and human (NES2Y) pancreatic beta-cells exposed to 1 μM and 10 μM concentrations of TDCIPP and TPhP for 1 week. Actin or HPRT was used as a loading control. The graphs represent means ± SEM from at least three independent sets of samples. Below each graph, a representative western blot is shown. **means statistical significance (*p* < 0.01) determined by ONE-WAYANOVA (Dunnet’s test)

Pancreatic beta-cells rely on cAMP signaling to enhance insulin secretion. Within these cells, cAMP molecules serve as activators of protein kinase A (PKA) and exchange protein directly activated by cAMP (Epac), both of which play essential roles in regulating the exocytosis mechanism involved in insulin release (Pratt et al. 2019). We tested the protein expression of active (autophosphorylated at Thr197) protein kinase A (p-PKA). None of the tested concentrations altered the p-PKA levels in beta-cells (Fig. 7A and 7C).

Activating transcription factor 3 (ATF3) is a cAMP-dependent transcription factor induced by various types of cellular stress, e.g., oxidative stress, ER stress, high glucose, or high fatty acids (Ku and Cheng 2020; Zmuda et al. 2010).

In human beta-cells, both 10 μM TDCIPP and 10 μM TPhP significantly decreased ATF3 protein expression: 10 μM TDCIPP to 43% of control and 10 μM TPhP to 47% of control (Fig. 7D).

### Enzymes involved in oxidative stress defense

Pancreatic beta-cells are known to be highly susceptible to oxidative stress due to their relatively lower levels of antioxidant enzymes compared to other cell types (Lenzen 2017). In our study, we measured the levels of three antioxidant enzymes: superoxide dismutase 1 (SOD1), glutathione reductase (Glu-Red), and biliverdin reductase B (BLVRB). SOD1 converts superoxide radicals into less harmful hydrogen peroxide and molecular oxygen. Glutathione reductase (Glu-Red) plays a crucial role in maintaining the levels of reduced glutathione, a vital antioxidant molecule in the cell. Biliverdin reductase B (BLVRB) is involved in the conversion of biliverdin into bilirubin, which possesses potent antioxidant properties and can help quench reactive oxygen species (Baranano et al. 2002; Sedlak and Snyder 2004).

**Fig. 8:**
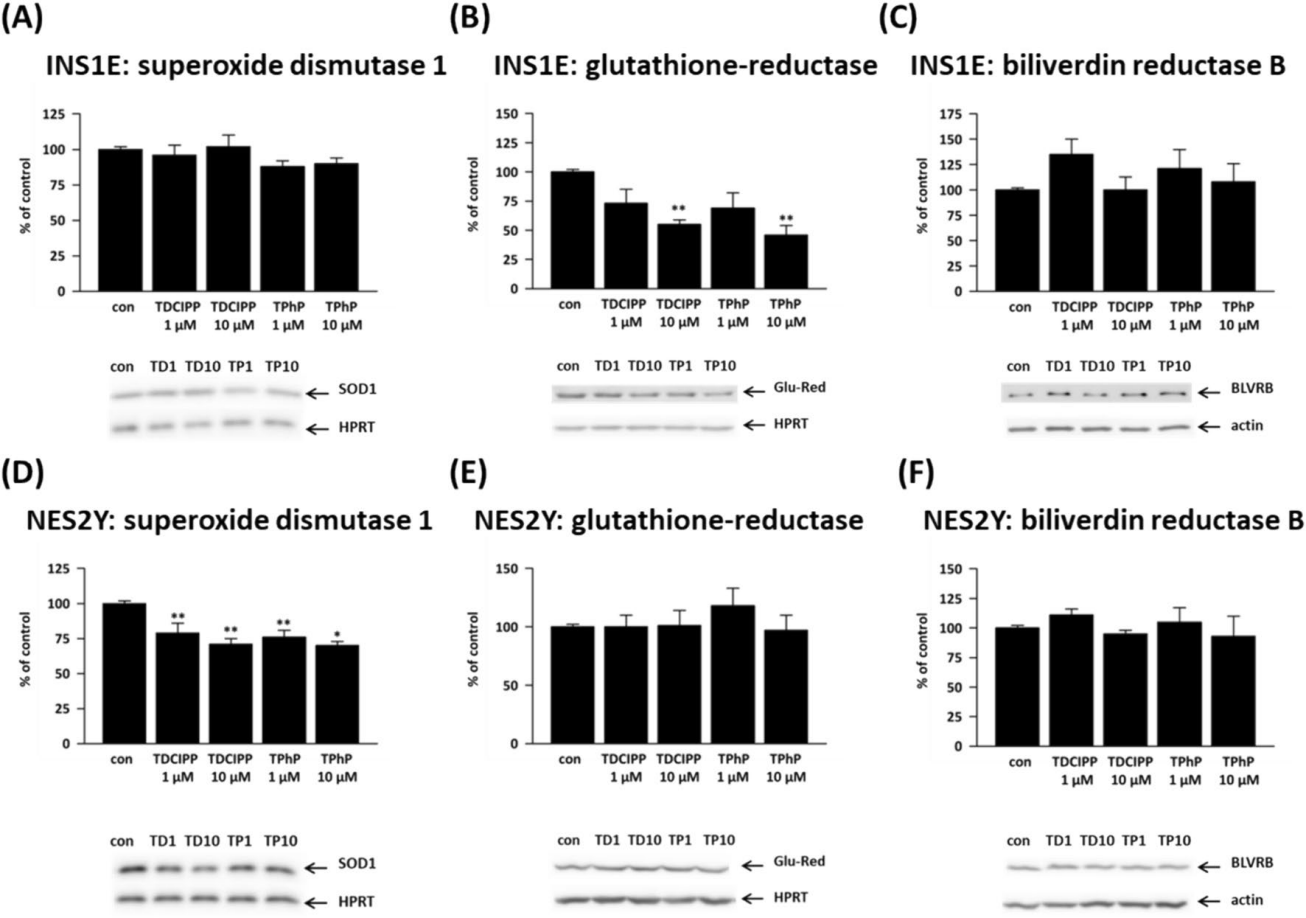
The protein expression of (**A**, **D**) superoxide dismutase 1 (SOD1), (**B**, **E**) glutathione reductase (Glu-Red), and (**C**, **F**) biliverdin reductase B (BLVRB) in the rat (INS1E) pancreatic beta-cells and human (NES2Y) pancreatic beta-cells exposed to 1 μM and 10 μM TDCIPP and TPhP for 1 week. Actin or HPRT was used as a loading control. The graphs represent an average of densitometric analyses of at least four western blots made of four independent sets of samples ± SEM. Below each graph, a representative western blot is shown. *means statistical significance (*p* < 0.05), **means statistical significance (*p* < 0.01), determined by ONE-WAYANOVA (Dunnet’s test)

All tested concentrations decreased protein expression of SOD1 in human beta-cells: 1 μM TDCIPP to 79% of control, 10 μM TDCIPP to 71% of control, 1 μM TPhP to 76% of control, and 10 μM TPhP to 70% of control (Fig. 8D). In rat beta-cells, SOD1 protein expression remained unchanged (Fig. 8A).

In human beta-cells, protein expression of glutathione reductase (Glu-Red) remained unchanged (Fig. 8E). In rat beta-cells, 10 μM TDCIPP significantly decreased Glu-Red expression to 60% of the control and 10 μM TPhP to 49% of the control (Fig. 8B).

None of the tested concentrations altered BLVRB (biliverdin reductase B) protein expression in beta-cells (Fig. 8C and 8F).

## Discussion

Our findings show that the exposure to TDCIPP and TPhP altered intracellular insulin levels and had an impact on other pancreatic beta-cell functions and homeostasis in both rat (INS1E) and human (NESY2) cell lines at non-cytotoxic concentrations (Table 1). More specifically, NADPH production, oxidative stress, glucose transport, ER stress, and lipid metabolism were impacted. These findings are in line with a few previous in vivo studies that reported the effects of TDCIPP and TPhP (in a mixture with tricresyl phosphate) on insulin levels and glucose clearance (Krumm et al. 2018; Walley et al. 2021). Altogether, these results raise the concern that exposure to these flame retardants could promote diabetes or obesity, calling for providing additional evidence.

**Table 1:**
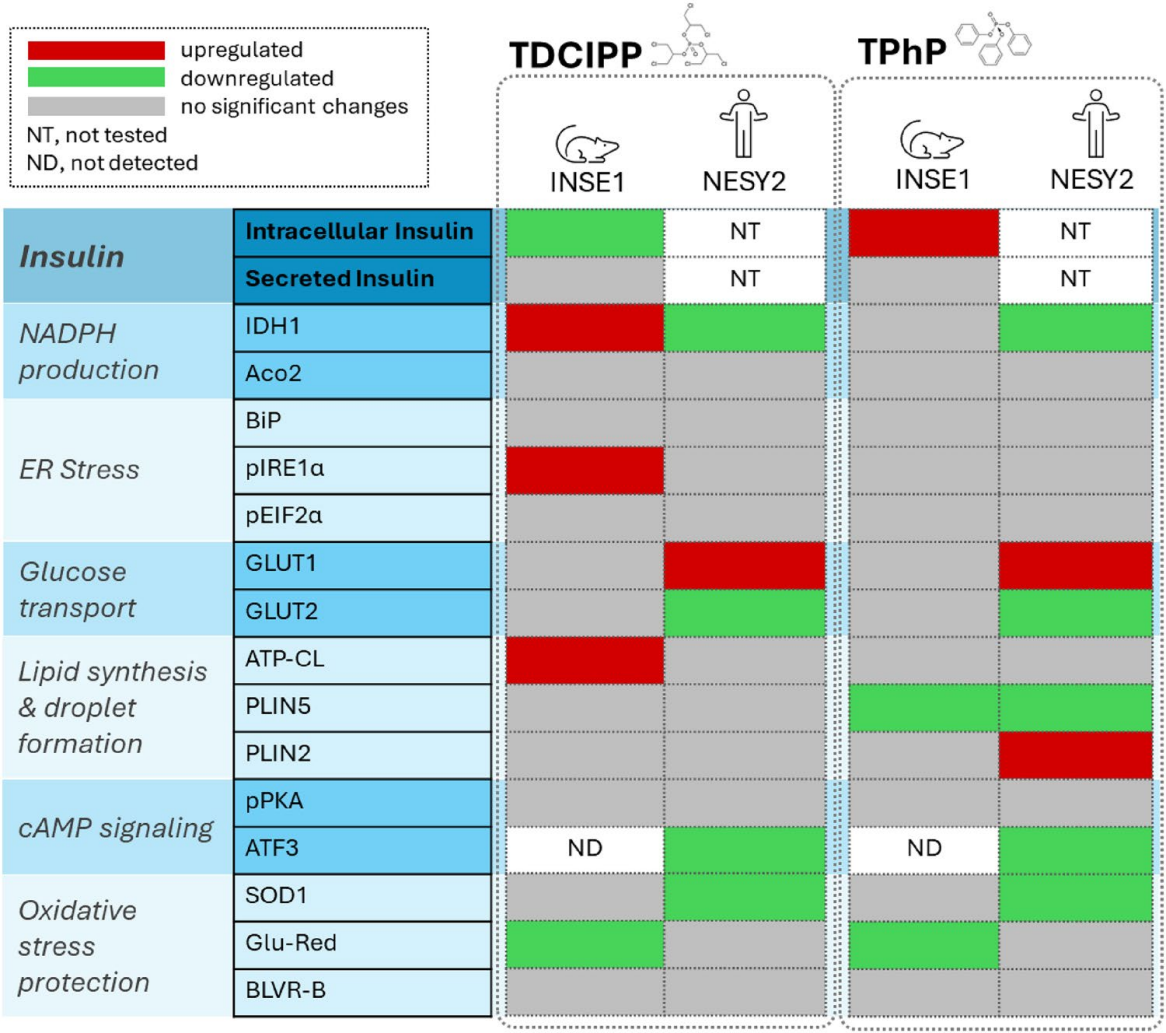
Summarizes the changes in protein expression in rat and human pancreatic beta-cell lines exposed to 10 μM TDCIPP and 10 μM TPhP for 1 week

Although both chemicals impacted pancreatic beta-cell lines, substantial differences existed between the effects of TDCIPP vs. TPhP, especially in rat beta-cells, with opposite effects on insulin production. In addition, differences existed between how each chemical affected INS1E and NESY2 cells. That may reflect a fundamental difference between rat and human species, highlighting the importance of including cell lines from different species when in vitro testing pancreatic beta-cell lines. These differences might also reveal disparities between cell lines with glucose-sensitive vs. constitutive insulin secretion. For example, the TDCIPP-induced increase in p-IRE1α was observed in rat beta-cells with glucose-stimulated insulin expression but not in human beta-cells with constitutive insulin secretion. In fact, the higher protein synthesis rate in cells with glucose-stimulated insulin expression might lead to a faster accumulation of unfolded proteins, making them more prone to ER stress than cells with constitutive insulin secretion. In the future, using glucose-sensitive human cell lines would be important. However, while glucose-sensitive rodent beta-cell lines exist (e.g., INS1E, INS/832, MIN6), establishing a glucose-sensitive human beta-cell line has been more challenging. The EndoC-betaH cell line is the only human glucose-sensitive beta-cell line available. Nevertheless, their cost, ease of use, and unfitness for long-term experiments limit their use.

Metabolites of both FRs are detected at high frequencies in human biomonitoring studies (Chen et al. 2018; Saillenfait et al. 2018; van der Schyff et al. 2023). The estimated daily intakes (EDIs) from levels of metabolites in urines or indoor dust ingestion range from 0.01 to 1560 ng/kg/day, with the highest values in children derived from studies in China and Latvia (Demirtepe et al. 2019; Ding et al. 2021; He et al. 2016; Chen et al. 2018; Li et al. 2019; Pasecnaja et al. 2021; Plichta et al. 2022). Estimating the human equivalent doses (HEDs) corresponding to the in vitro concentrations used in this study requires quantitative in vitro-to-in vivo extrapolation (Najjar et al. 2022). A recent study using such an approach calculated that 5 µM effective doses in in-vitro hepatic cell models would correspond to approximately 0.2 (for TDCIPP) and 0.6 (for TPhP) mg/kg/d in children, which are several orders of magnitude higher than the human EDIs (Ding et al. 2021). On the other hand, the OPFRs plasma concentrations in µM estimated from indoor dust using a high-throughput toxicokinetic model range from 0.003 to 32 µM for TDCIPP and from 0.00006 to 1.4 µM for TPhP (Blum et al. 2019a). Therefore, the concentrations used in our study would be in the range of the highest estimated plasma concentrations. However, caution should be taken when comparing the nominal concentrations used in vitro with real-life exposures since it relies on model-based estimates, and other factors may also influence. For instance, the concentration of free chemicals in the medium may differ from the nominal concentration due, for example, to sorption to components of the in vitro setup (e.g., plastic and serum) (Groothuis et al. 2019). In addition, in our study, the exposure is limited to 1 week while the human population is potentially exposed chronically to these flame retardants.

### Effect of TDCIPP and TPhP in rat beta-cells

TDCIPP decreased the intracellular insulin and proinsulin levels in the rat beta-cell line. The TDCIPP-induced increase in ER stress might contribute, at least partially, to the decrease in insulin production observed following exposure to TDCIPP. Indeed, ER stress in pancreatic beta-cells leads to a sudden drop in insulin production and the manifestation of type 2 diabetes mellitus (Burgos-Morón et al. 2019; Morikawa and Urano 2023).

However, in cells exposed to 10 μM TDCIPP, the reduced intracellular insulin levels did not lead to a decrease in secreted insulin levels at the time examined. In our study, we detected effects on several proteins that might increase insulin secretion, compensating for the decrease in insulin production and normalizing the levels of secreted insulin. For instance, the increase in IDH1 and the decrease in Glu-Red may elevate the levels of NADPH. Indeed, IDH1 inhibition has been shown to suppress insulin secretion in wild-type mouse islets (Bauchle et al. 2021). Glu-Red is highly expressed in pancreatic islet cells (Nagaoka et al. 2004), where it produces reduced glutathione needed for oxidative stress defense but also consumes NADPH needed for the amplification of insulin secretion. Its expression (Guo et al. 2013) and activity (Xiao et al. 2023) were decreased in the diabetic mice pancreases and MIN6 mouse beta-cells exposed to a high glucose concentration (Xu et al. 2023). In addition, the decreasing trend of aconitase-2 expression, albeit statistically insignificant, may contribute to the overall impact on NADPH production by reducing the substrate level for IDH1. Another potential mechanism involves the increased expression of ATP-CL that may stimulate insulin secretion through malonyl-CoA production (Aghelan et al. 2020). Indeed, Flamez and coworkers showed that ATP-CL inhibitors radicicol and (-)-hydroxy-citrate blocked part of glucose-stimulated insulin secretion in rat beta-cells (Flamez et al. 2002). Therefore, the elevated levels of IDH1, the reduction of Glu-Red levels, and the increased expression of ATP-CL in rat beta-cells exposed to 10 μM TDCIPP may work in concert to normalize insulin secretion despite the decrease in intracellular insulin levels. In such a scenario, longer-term or chronic exposures to TDCIPP might decrease the levels of secreted insulin after the levels of intracellular insulin are too low or depleted.

Contrary to TDCIPP, TPhP exposure increased intracellular proinsulin and insulin levels in INS1E cells. We did not detect significant changes in the levels of secreted insulin in cells exposed to 10 μM TPhP, but an increasing trend was consistent with the effect on insulin production. The decreased levels of Glu-Red might potentially contribute to an increase in insulin secretion by elevating the levels of NADPH. Excessive insulin production can lead to a more rapid decline in blood glucose levels, triggering hunger. Consequently, compounds exhibiting such effects have the potential to promote obesity. Obesity as a result of hyperinsulinemia was previously described, e.g., in mice overproducing glutathione peroxidase 1 (Wang et al. 2008). Interestingly, unlike in macrophages and hepatocytes (Hu et al. 2020; Yue et al. 2023), TPhP failed to induce ER stress in beta-cells, suggesting differential effects on ER stress induction in various cell types. The exposure to 10 μM TPhP also decreased PLIN5 expression in rat beta-cells. PLIN5 plays a dual role in lipid metabolism by participating in lipid droplet formation while, under certain conditions, also promoting lipolysis and fatty acid oxidation to meet metabolic demands (Zhu et al. 2019). Moreover, in INS1E cells, overexpression of PLIN5 has been shown to protect against palmitic acid-induced toxicity (Zhu et al. 2019). This protective effect is attributed to PLIN5’s ability to decrease endoplasmic reticulum (ER) stress (Zhu et al. 2019) and increase the expression of Nrf2, a key regulator of cellular antioxidant response (Zhu et al. 2020). By reducing PLIN5 expression, TPhP may disrupt the balance of lipid metabolism and impair beta-cells’ ability to handle excessive fatty acids, increasing their susceptibility to lipotoxic damage. Several studies have described the effects of TPhP exposure on fat metabolism. 10 μM TPhP increased lipid accumulation in liver cells (Negi et al. 2021), adipogenic differentiation and lipolysis in 3T3-L1 adipocytes (Cano-Sancho et al. 2017) and induced total cholesterol and total triglyceride accumulation in HepG2 cells (Hao et al. 2019). However, it is important to note that lipid droplet formation likely has a beneficial function in beta-cells (Sramek et al. 2021). In the case of fatty acid overload, beta-cells utilize lipid droplets to sequester fatty acids, preventing them from inducing toxicity (Tong et al. 2022; Zheng et al. 2022).

### Effect of TDCIPP and TPhP in human beta-cells

The effects on insulin production and secretion could not be measured in the human beta-cell line, but TDCIPP and TPhP impacted glucose transporters, IDH1, ATF3, and SOD1. In addition, both TDCIPP and TPhP increased GLUT1 expression while decreasing GLUT2 expression. In rodent beta-cells, the primary glucose transporter responsible for glucose uptake into the cells is GLUT2 (Mueckler and Thorens 2013). Interestingly, the islets isolated from GLUT2-null mice demonstrated impaired glucose-stimulated insulin secretion and synthesis, highlighting the significance of GLUT2 in these processes (Guillam et al. 2000). Although similar experiments in human pancreatic beta-cells are not available, research by McCulloch et al. suggests that GLUT1, rather than GLUT2, is primarily responsible for transporting glucose into human beta-cells (McCulloch et al. 2011). Therefore, the increase in GLUT1 levels might have a more significant impact than the decrease in GLUT2 and it is plausible to expect an overall increase in glucose transport in human beta-cells exposed to TDCIPP or TPhP.

In human beta-cells, all four exposure conditions significantly decreased SOD1 levels. The decreased SOD1 levels could increase the vulnerability of pancreatic beta-cells against superoxide. Interestingly, SOD1-knockout mice showed decreased insulin secretion (Muscogiuri et al. 2013). Together with the decrease in IDH1, the decrease in SOD1 might, therefore, contribute to reduced insulin secretion in human cells.

The exposure to 10 μM TCDIPP and 10 μM TPhP also negatively affected ATF3 levels in human beta-cells. Studies in rodents suggest that ATF3 is required for proper insulin production in response to a fatty diet, having a rather protective effect on pancreatic beta-cells (Zmuda et al. 2010). When exposed to a high-fat diet, mice with a knockout ATF3 gene had significantly reduced serum insulin levels compared to wild-type mice (Zmuda et al. 2010). In human beta-cells, TDCIPP- and TPhP-induced decrease in ATF3 levels may potentially diminish the defense mechanisms of these cells against various adverse conditions. However, further research is needed to fully understand the role of ATF3 in stress defense and its implications for human beta-cell function and insulin secretion.

TPhP also impacted perilipins in human cell lines, decreasing PLIN5 expression while increasing PLIN2. PLIN2, also known as adipose differentiation-related protein (ADRP), is primarily associated with lipid droplets and is expressed in various tissues, including pancreatic beta-cells (Tong et al. 2022). However, its overexpression in beta-cells appears to have negative implications. Research conducted by Chen and coworkers (Chen et al. 2017) demonstrated that in mouse islets and the mouse beta-cell line MIN6, overexpression of PLIN2 led to increased endoplasmic reticulum (ER) stress when exposed to lipid overload or chemical ER stress inducers. Conversely, silencing of PLIN2 alleviated this effect, suggesting a detrimental role of PLIN2 in promoting ER stress (Chen et al. 2017). Additionally, elevated expression of PLIN2 was observed in human islets obtained from donors with type 2 diabetes mellitus (Tong et al. 2022). The overexpression of PLIN2 in human beta-cells exposed to 10 μM TPhP likely contributes together with PLIN5 downregulation to an imbalance in lipid handling and storage within beta-cells, potentially leading to disturbances in lipid homeostasis. This disruption in lipid balance can have detrimental consequences for beta-cell function and insulin secretion when facing saturated fatty acids or nutritional overload.

## Conclusions

Our findings indicate that the exposure to TDCIPP and TPhP impacted pancreatic beta-cell functions and homeostasis in both rat (INS1E) and human (NESY2) cell lines. Notably, both chemicals altered intracellular insulin and proinsulin levels in rat cells. Although we did not observe significant changes in the secreted insulin levels, we theorize that more extended exposure periods exceeding one week may eventually impact insulin secretion. Insulin levels could not be measured in human cells, but the effects on NADPH production, glucose transport, or oxidative stress in human and rat cells might alter insulin production and secretion. The alterations in protein expression, as shown in Table 1, indicate that exposure to flame retardants negatively disrupted beta-cell homeostasis, potentially rendering the beta-cells more susceptible to adverse conditions such as excessive nutrient intake or oxidative stress.

We compared the effects of flame retardants on two different beta-cell lines, rat and human. In most cases, TDCIPP and TPhP induced significant perturbations to both beta-cell lines but with substantial differences in the proteins/pathways affected. That might reflect important differences between rat and human beta-cells, as described before (Benner et al. 2014; Klemen et al. 2017; Law et al. 2010; McCulloch et al. 2011) and/or between cells with glucose-sensitive and constitutive insulin secretion. That highlights the relevance of using cells from both species, ideally glucose-sensitive, when testing chemicals.

Currently, there is a lack of systematic testing for the potential pro-diabetic effects of industrial chemicals before they are introduced to the market. Our results highlight the importance of focusing more on pancreatic cells and diabetes in toxicological studies. Given the ongoing diabetes epidemic, it would greatly benefit public health to develop an in vitro-based test battery that assesses the toxicity of chemicals on pancreatic beta-cells prior to widespread exposure in the population.

## Data Availability

The complete data supporting the findings present in the article can be provided by the corresponding author upon a reasonable request.
